# Are the immunomodulatory properties of *Lactobacillus rhamnosus* CRL1505 peptidoglycan common for all Lactobacilli during respiratory infection in malnourished mice?

**DOI:** 10.1371/journal.pone.0194034

**Published:** 2018-03-08

**Authors:** Yanina Kolling, Susana Salva, Julio Villena, Susana Alvarez

**Affiliations:** 1 Laboratory of Immunobiotechnology, Reference Centre for Lactobacilli (CERELA-CONICET), Tucuman, Argentina; 2 Institute of Applied Biochemistry, Tucuman University, Tucuman, Argentina; Universite Clermont Auvergne, FRANCE

## Abstract

Previously, we reported that *Lactobacillus rhamnosus* CRL1505 peptidoglycan (PG05) improves the innate immune response in immunocompromised-malnourished mice after *Streptococcus pneumoniae* infection. This study extends those previous findings by demonstrating that the dietary recovery of malnourished mice with nasal administration of PG05 improves not only the innate immune response but the respiratory and systemic adaptive humoral response as well. PG05 enhanced the Th2 response, the recovery of B cells, and the concentration and opsonophagocytic activity of anti-pneumococcal antibodies. In addition, by performing comparative studies with the peptidoglycans from lactobacilli of the same species (*L*. *rhamnosus* CRL534) or with similar immunomodulatory properties (*L*. *plantarum* CRL1506), we demonstrated here that PG05 has unique immunomodulatory properties that cannot be extended to peptidoglycans from other probiotic strains. However, the knowledge of the molecular characteristics of PG05 is indispensable to understand immunomodulatory abilities of *L*. *rhamnosus* CRL1505.

## Introduction

Probiotic microorganisms able to regulate the immune system (immunobiotics) are generally selected from lactic acid bacteria (LAB) strains [1]. It is now well established that immunobiotics are capable to interact with the host's immune and non-immune cells, and thereby impact on mucosal and systemic immune responses [2–4]. It has been suggested that the final response of the host to an immunobiotic bacteria depends on the combination of the different bacterial molecules that can interact with the several receptors located in host´s cells [4]. Moreover, over the last years several molecules from immunobiotic bacteria have been associated to the immunomodulatory effects and their beneficial impact on health including cell wall, peptidoglycan, exopolysaccharides and secreted metabolites [5–7].

*Lactobacillus rhamnosus* CRL1505 is an immunobiotic strain that has been extensively studied by our laboratory because of its outstanding capacity to improve mucosal defenses in both immunocompetent and immunocompromised hosts [2,3]. By using a mice model of immunosuppression associated to protein malnutrition, we demonstrated that malnourished hosts are highly susceptible to respiratory pneumococcal infection [8,9]. The diminished resistance to *Streptococcus pneumoniae* infection in malnourished mice was associated with multiple abnormalities of the immune system including alterations in emergency granulopoiesis, phagocyte function, antibodies and cytokine production, B cell development, and CD4 Th2 cells numbers and activities [8–12]. Of note, we found that the supplementation of balance conventional diet (BCD) with nasally or orally administered *L*. *rhamnosus* CRL1505, during the recovery of malnourished mice, was able to improve respiratory innate and humoral immune responses and reduce the susceptibiltiy to pneumococcal infection [10,11,13]. While the viability of immunobiotic lactobacilli is an important factor to achieve optimal protective effects, it is possible to stimulate immunity using non-viable immunobiotics [14,15]. Therefore, administration of non-viable immunobiotics or their cellular components to beneficially modulate immune responses represents an interesting and safe alternative specially in immunocompromised hosts [16]. In this regard, we have reported that non-viable immunobiotics were effective in the modulation of the respiratory and systemic immune system in malnourished hosts under repletion treatments [14,15]. Moreover, we also found that the peptidoglycan was able to preserve the immunomodulatory capacity of viable *L*. *rhamnosus* CRL1505, and was capable to improve the resistance against pneumococcal infection in immunocompromised malnourished mice by enhancing the recovery of the innate immune response [15].

We hypothesized that: a) the peptidoglycan of *L*. *rhamnosus* CRL1505 is as effective as the viable strain for improving not only the innate but also the adaptive immune response against *S*. *pneumoniae* in malnourished mice, and b) the immunomodulatory properties of *L*. *rhamnosus* CRL1505 peptidoglycan in malnourished mice are specific and are not common to peptidoglycans of other lactobacilli strains. Therefore, in this this work we performed a comparative study to evaluate the effect of peptidoglycans from different lactobacilli on the innate and adaptive immune responses to pneumococcal infection in malnourished mice.

## Materials and methods

### Lactobacilli and peptidoglycans

*L*. *rhamnosus* CRL1505, *L*. *plantarum* CRL1506 and *L*. *rhamnosus* CRL534 were obtained from the CERELA culture collection (Chacabuco 145, San Miguel de Tucumán, Argentina). The cultures were kept freeze-dried and then rehydrated using the following medium: peptone 15.0 g, tryptone 10.0 g, meat extract 5.0 g, distilled water 1 l, pH 7. Lactobacilli were cultured for 18 h at 37°C (final log phase) in Man- Rogosa-Sharpe broth (MRS, Oxoid). Bacteria were harvested by centrifugation at 3000 g for 10 min, washed three times with sterile 0.01 mol/l phosphate buffer saline (PBS), pH 7.2, and resuspended in sterile on PBS. Peptidoglycans from the three strains were obtained using the method of Shida *et al*. [17] with modifications, as we previously described [15]. In all cases, the peptidoglycan preparations were free of contaminants such as lipoteichoic acid, wall teichoic acid and nucleic acid, as phosphorus was below the level of detection (< 10 nmol/mg) in the preparation. We performed a study using Fourier Transform Infrared Spectroscopy (FT-IR). The assignment of bands and the similarity exhibited by the spectral profiles among them indicated that the samples would be peptidoglycans isolated from lactic acid bacteria.

### Animals and feeding procedures

Male 3-week-old Swiss-albino mice were obtained from CERELA. Malnourished mice were obtained from weaned mice that were were fed a protein-free diet (PFD) for 21 days as previously described [15]. Malnourished mice were divided in four groups for treatments: repletion with a balanced conventional diet (BCD) during 7 days (BCD control group); repletion for 7 d with BCD and nasally adminsitered *L*. *rhamnosus* CRL1505 (PG05 group), *L*. *plantarum* CRL1506 (PG06 group), or *L*. *rhamnosus* CRL534 (PG534 group) peptidoglycans in the last 2 days. Each peptidoglycan was administered in a dose of 8 μg/ml.

The doses of peptidoglycans were chosen on the basis of our previous results [15]. The compositions of the BCD and PFD diets were described previously [8]. Animal protocols were approved by the Ethical Animal Protection Committee of CERELA-CONICET, Tucumán, Argentina, under the protocol number BIOT-CRL-10, and all experiments comply with the current laws of Argentina and all international organizations for the use of experimental animals.

### *Streptococcus pneumoniae* and pneumococcal infection

*Streptococcus pneumoniae* serotype 6B (one of the 10 most frequent serotypes isolated from pneumococcal infections in Argentina) was isolated from the respiratory tract of a patient from the Department of Bacteriology of Malbran Institute (Buenos Aires, Argentina). Serotypification was carried out at the Administración Nacional de Laboratorios e Institutos de Salud (Buenos Aires, Argentina).

Challenge with *S*. *pneumoniae* was carried out at the end of each feeding procedure by dropping 25 μL of the inoculum containing 10^9^ log-phase cells of *S*. *pneumoniae* in PBS into each nostril and involuntarily inhaled [15]. To facilitate migration of the inoculum to the alveoli, mice were held in a vertical position for 2 min. Animals were sacrificed at day 0 (before challenge) and at different days post-infection.

### Bacterial cell counts in lung and blood

For the evaluation of *S*. *pneumoniae* infection, lungs were excised, weighed and homogenized in 5 ml of sterile 0.1% peptone water. Homogenates were diluted appropriately, plated in duplicate on blood agar and incubated 18 h at 37°C. The results were expressed as log of CFU/g lung. Progression of bacterial growth into the bloodstream was monitored by sampling blood obtained through cardiac puncture and plating on blood agar. Results were reported as log of CFU/ml [15].

### Lung injury

For the evaluation of pulmonary tissue injuries, lungs were excised, washed out with PBS, and immersed in 4% (v/v) formalin saline solution for the histologycal study as previously mentioned [15]. In addition, biochemical parameters as albumin content and lactate dehydrogenase (LDH) activity were studied in broncho-alveolar lavages (BAL). Albumin content was determined colorimetrically based on albumin binding to bromocresol green using an albumin diagnostic kit (Wiener Lab). LDH activity was determined by measuring the formation of the reduced form of nicotinamide adenine dinucleotide using the Wiener reagents and procedures (Wiener Lab), and was expressed as units per liter of BAL fluid [18].

### Leukocyte counts in blood and BAL

Blood samples were obtained by cardiac puncture at the end of each treatment and collected in heparinized tubes. BAL samples were obtained as described previously [15]. Briefly, the trachea was exposed surgically and intubated with a catheter. A small incision was made in the trachea and two sequential bronchoalveolar lavages were performed in each mouse by injecting sterile PBS with 1% heparin. The recovered fluid was centrifuged for 10 min at 300 rpm. The pellet was used to perform the leukocyte count following the conventional hematological methodology. Total number of blood and BAL leukocytes was determined with a hemocytometer. Differential cell counts were performed by counting 200 cells in blood and BAL smears stained with May-Grünwald Giemsa using a light microscope (1000×), and absolute cell numbers were calculated [19].

### Myeloperoxidase activity of neutrophils

Measurement of myeloperoxidase (MPO) activity of blood neutrophils was carried out by the use of the Washburn test, which is a cytochemical method that uses benzidine as an MPO chromogen [9]. Cells were graded as negative or weak, moderate, or strongly positive according to the intensity of reaction and were used to calculate the score. The score was calculated by counting 200 neutrophils in blood smears. The score value was calculated by the addition of neutrophils with different positive grades.

### Cytokines and antibodies in serum and BAL

Tumour necrosis factor (TNF)-α, interleukin (IL)-10, IL-2, IL-4, and Interferon (INF)-γ were measured in serum and BAL samples at days 2 and/or 10 post-infection by using commercially available enzyme-linked immunosorbent assay (ELISA) technique kits, following the manufacturer's recommendations (ELISPOT Ready-SET-Go!®, eBioscience, San Diego, USA). Anti-pneumococcal antibodies (IgA, IgM, and IgG) were determined by ELISA on day 10 post-infection as previoulsy described [8]. In brief, plates were coated with a 1:100 dilution of heat-killed *S*. *pneumoniae* overnight at 4°C and blocked with PBS containing 5% non-fat dry milk. Appropriate dilutions of the samples (serum 1:20; BAL 1:2) were incubated for 1 h at 37°C. Peroxidase conjugated anti-mouse IgG, IgA, or IgM (1:500) (Sigma- Aldrich) were added and incubated for 1 h at 37°C. The reaction was developed with TMB Substrate Reagent (Sigma-Aldrich). The concentration was measured with reference to standard curves using known amounts of the respective murine Immunoglobulin (Sigma-Aldrich).

### Opsonophagocytic activity

Heat-killed pneumococci were incubated with FITC (Fluorescein 5(6)-isothiocyanate, Sigma-Aldrich) (15 ug/ml) for 30 min at 37°C), and then washed three times with PBS buffer (pH 7.4). The opsonophagocytic activity of BAL and serum antibodies was determined by measuring the phagocytosis of FITC-labeled pneumococci by peritoneal macrophages. For this purpose, FITC-labeled *S*. *pneumoniae* was incubated with BAL or serum samples at 37°C for 30 min. The opsonization mix was the added to 10^6^ macrophages/ml (macrophage/bacteria ratio of 1/100) and incubated for 30 min. Phagocytosis was measured by flow cytometry. The external fluorescence was blocked with the addition of trypan blue to the cell suspensions. The difference between blocked and non-blocked samples was calculated and represented in MFI graph [20,21].

### Flow cytometry studies

Single cell suspensions of spleen, thymus and lung were prepared according to Barbieri et al. [10]. Lungs were removed, incubated for 60 min with 300 U of Type I collagenase (Sigma-Aldrich) in 3 ml of RPMI 1640 medium (Sigma-Aldrich) and then minced into small pieces. To dissociate the tissue into single cells, collagenase-treated minced lungs were gently tapped into a plastic dish. After removal of debris, erythrocytes were depleted by hypotonic lysis (Tris-ammonium chloride, BD PharMingen). The cells were washed and resuspended in RPMI supplemented with 4% heat-inactivated foetal calf serum (FCS). Spleens and thymus were collected and tissue was homogenized through a tissue strainer with PBS 1% FCS, followed by incubation with lysis buffer to eliminate erythrocytes [22]. Bone marrow cells were obtained by flushing femurs of mice using cold PBS 1% FCS. Cell suspensions were centrifuged 4 min at 2500 rpm at 4°C, and incubated with lysis buffer to eliminate erythrocytes [22]. Cell viability was assessed by Trypan blue exclusion. Cells were kept on ice until immunofluorescence labeling.

The following antibodies from BD PharMingen were used: FITC labeled anti-mouse-CD3, biotinylated anti-mouse-CD4, PE labeled anti-mouse-CD8, FITC labeled anti-mouse-CD11c, PE labeled anti-mouse-MHCII, APC labeled anti-mouse- CD45, PE labeled anti-mouse-Gr-1, APC labeled anti-mouse-F_4/80_, FITC labeled anti-mouse-CD25, biotinylated anti-mouse-TCR_αβ_, biotinylated anti-mouse-B220, FITC labeled anti-mouse-IgM, and biotinylated anti-mouse-IgD antibodies. Following incubation with biotinylated primary antibodies, the labeling was revealed using streptavidin-PercP. In all cases, cells were acquired on a BD FACSCalibur™ flow cytometer (BD Biosciences) and data was analyzed with FlowJo software (TreeStar). The total number of cells in each population was determined by multiplying the percentages of subsets within a series of negative markers or positive gates by the total cell number determined for each tissue.

### Statistical analysis

Statistical analysis was performed using GraphPad Prism 6.0 software. Experiments were performed in triplicate and the results were expressed as mean ± standard deviation (SD). After verification of the normal distribution of data, 2-way ANOVA was used. Tukey's test (for pairwise comparisons of the means) was used to test the differences between the groups. Differences were considered significant at p<0.05, p<0.01, or p<0.001.

## Results

### Effect of lactobacilli peptidoglycans on the resistance to pneumococcal infection in malnourished mice

In previous reports we demonstrated that malnourished mice have an increased susceptibility to pneumococcal infection [8,9] and that the repletion with BCD supplemented with nasally administered PG05 significantly increase the resistance against the respiratory pathogen [15]. In this work, we first aimed to investigate whether the peptidoglycans obtained from different lactobacilli strains were able to induce a similar effect. Therefore, we comparatively studied the effect of PG05 and peptidoglycans from *L*. *plantarum* CRL1506 (PG06, immunomodulatory strain), and *L*. *rhamnosus* CRL534 (PG534, non-immunomodulatory strain). As we previously observed, repletion of malnourished mice with BCD supplemented with the nasal administration of PG05 allowed the elimination of the pathogen since it was not detected in lung or blood (Table 1). On the contrary, PG06 and PG534 treatments failed to induce this effect since pneumococcal cell counts in lungs and blood were similar to those observed in the BCD control group (Table 1).

**Table 1:** Resistance to pneumococcal infection.

	Lung	Blood
**BCD**	4.47± 0.05 *	5.15± 0.10 *
**BCD+PG05**	< 1.5	< 1.5
**BCD+PG06**	4.47± 0.08 *	5.14± 0.12 *
**BCD+PG534**	4.62± 0.08 *	5.28± 0.04 *

Malnourished mice were replete for 7 days with a balanced conventional diet (BCD) or BCD supplemented with nasal administration of peptidoglycans from *Lactobacillus rhamnosus* CRL1505 (BCD+PG05), *Lactobacillus plantarum* CRL1506 (BCD+PG06) or *Lactobacillus rhamnosus* CRL534 (BCD+PG534), and then challenged with *S*. *pneumoniae*. Bacterial cell counts in lung (log CFU/g of lung) and blood (CFU/ ml) after challenge. Data are for six individual mice per group. Results represent data from three independent experiments. Results are expressed as mean ± SD. Asterisks represent statistical differences compared with BCD+PG05 group.

* p ≤ 0.001.

**Fig 1:**
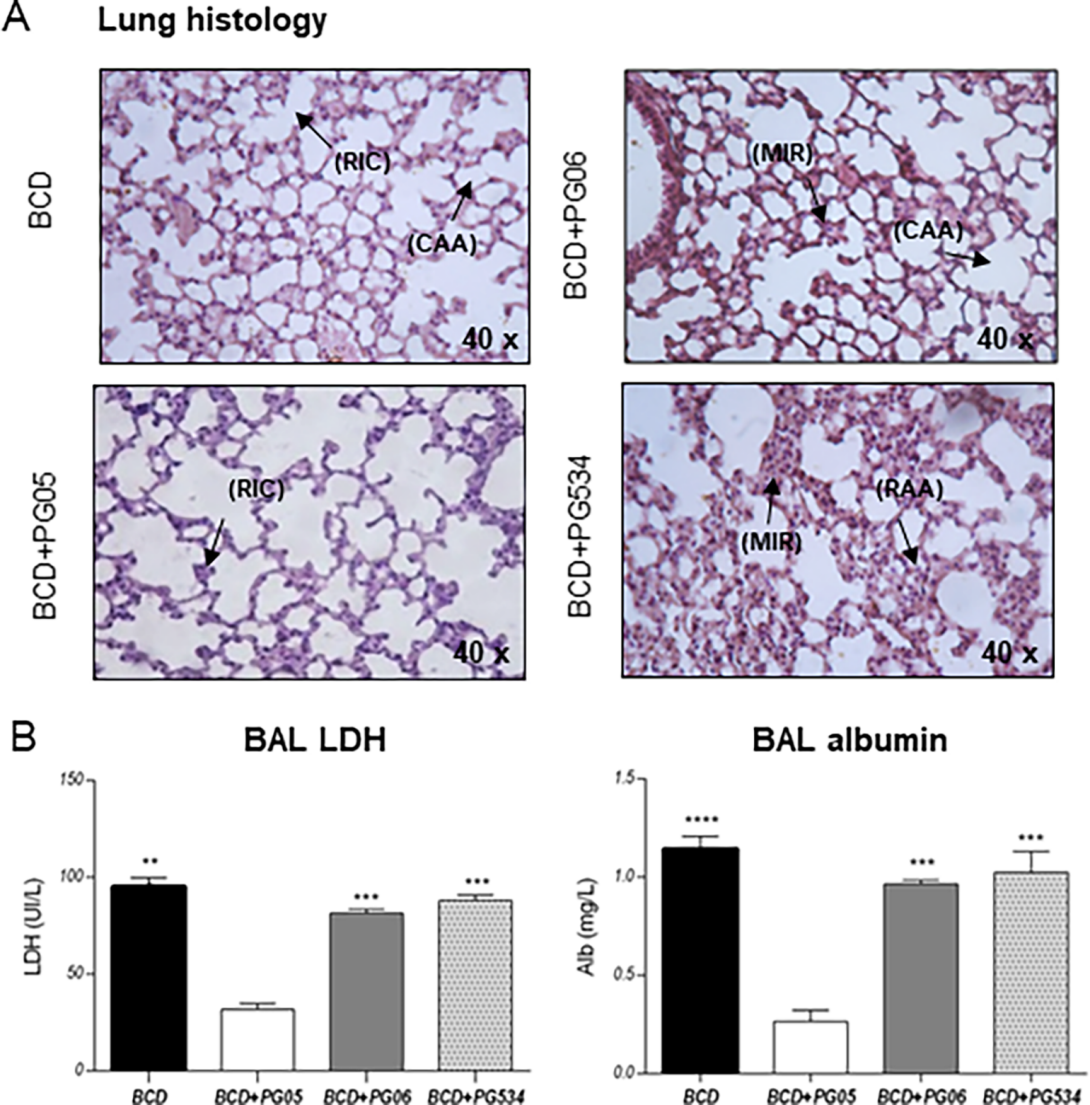
Effect of lactobacilli peptidoglycans on lung injuries induced by the infection of *Streptococcus pneumoniae* in malnourished mice. Immunocompromised-malnourished mice were replete for 7 days with a balanced conventional diet (BCD) or BCD supplemented with peptidoglycans from *Lactobacillus rhamnosus* CRL1505 (BCD+PG05), *L*. *plantarum* CRL1506 (BCD+PG06) or *L*. *rhamnosus* CRL534 (BCD+PG534), and then challenged with *S*. *pneumoniae*. Two days after pneumococcal challenge, lung damage was evaluated by histological studies and biochemical parameters. (A) Lungs histological examination. Hematoxylin and eosin stained light micrographs, original magnification ×100. Recruited inflammatory cells (RIC), reduction of alveolar airspaces (RAA), moderate inflammatory response (MIR), conserved alveolar air spaces (CAA). (B) Lactate dehydrogenase (LDH) activity and, albumin concentration in broncho-alveolar lavages (BAL). The results represent data from three independent experiments. Results are expressed as mean ± SD. Asterisks represent statistical differences compared with the BCD+PG05 group. * p < 0.005, ** p ≤ 0.001.

We also evaluated lung tissue injury by performing histological studies and determining BAL albumin content, a measure of the bronchoalveolar–capillarity barrier permeability, and BAL LDH activity, an indicator of general cytotoxicity (Fig 1). The infectious challenge with *S*. *pneumoniae* induced a moderate infiltration of inflammatory cells in the lungs of BCD and BCD+PG06 groups. BCD+PG534 group showed a prominent infiltration of inflammatory cells into the lung parenchyma. In addition, a preserved lung parenchyma and reduced cell infiltration was observed in BCD+PG05 group (Fig 1A). Levels of both LDH and albumin in BAL were higher in BCD, BCD+PG06, and BCD+PG534 groups when compared to those observed for the BCD+PG05 treatment (Fig 1B).

### Effect of lactobacilli peptidoglycans on the innate immune response to pneumococcal infection in malnourished mice

**Fig 2:**
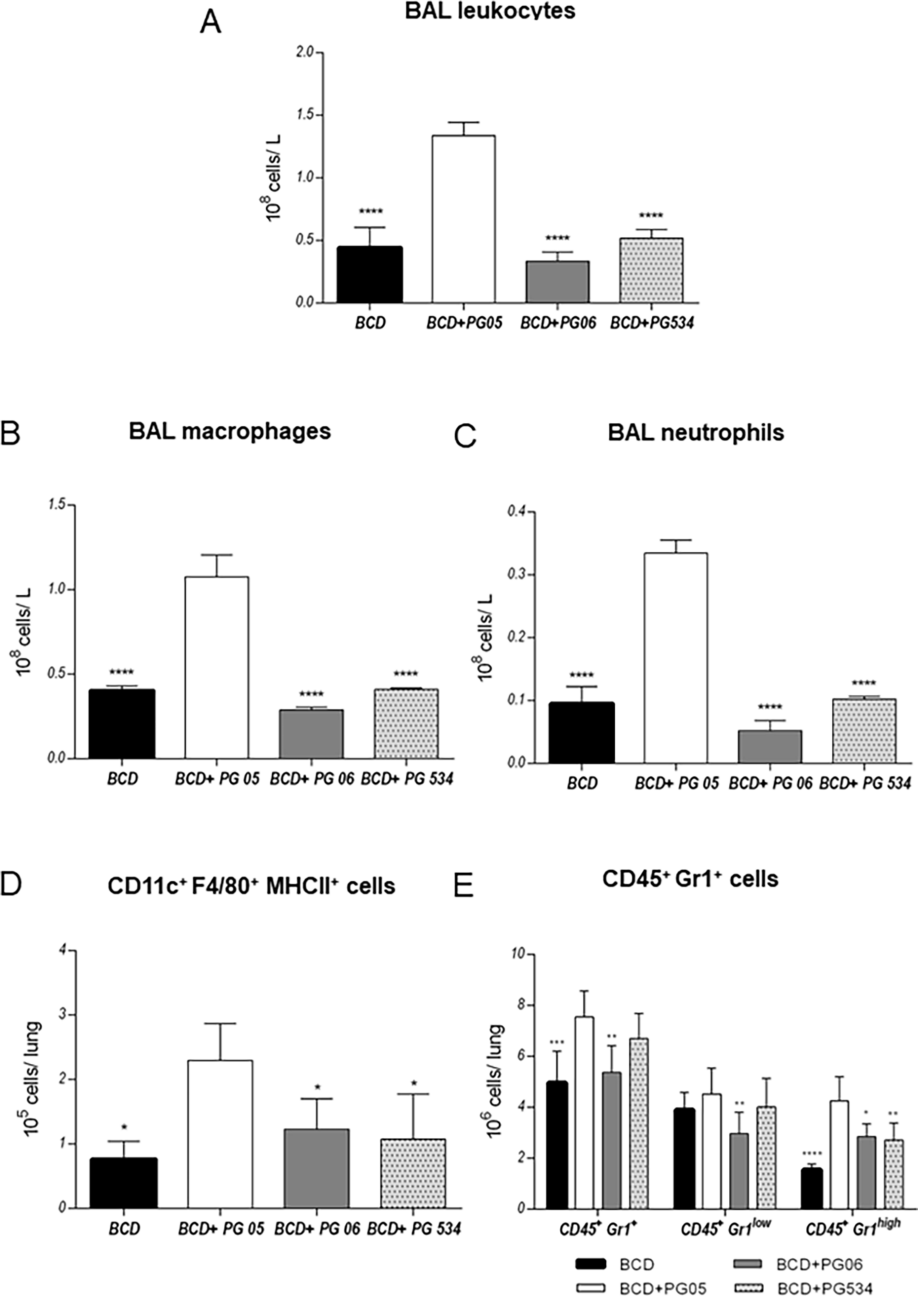
Effect of lactobacilli peptidoglycans on the respiratory innate immune response to *Streptococcus pneumoniae* infection in malnourished mice. Immunocompromised-malnourished mice were replete for 7 days with a balanced conventional diet (BCD) or BCD supplemented with peptidoglycans from *Lactobacillus rhamnosus* CRL1505 (BCD+PG05), *L*. *plantarum* CRL1506 (BCD+PG06) or *L*. *rhamnosus* CRL534 (BCD+PG534), and then challenged with *S*. *pneumoniae*. The respiratory innate immune response was studied two days after the pneumococcal challenge. (A) Number of broncho-alveolar lavages (BAL) leukocytes. (B) Number of BAL macrophages. (C) Number of BAL neutrophils. (D) Number of lung macrophages (CD11c^+^F480^+^MHCII^+^). (E) Number of lung neutrophils (CD_45_^+^Gr1^+^). Results represent data from three independent experiments. Results are expressed as mean ± SD. Asterisks represent statistical differences compared with BCD+PG05 group. * p < 0.05, ** p ≤ 0.001, ***/**** p ≤ 0.001.

We next studied the effect of the different peptidoglycans on the respiratory and systemic innate immune response by evaluating the number of lung and blood leukocytes and the levels of respiratory and systemic cytokines. As we reported previously [15], the challenge with *S*. *pneumoniae* significantly increased the number of BAL leucocytes in all the experimental groups (Fig 2A–2C). However, levels of BAL leucocytes (Fig 2A), macrophages (Fig 2B), and neutrophils (Fig 2C) were significantly higher in BCD+PG05-treated mice than in the other groups. In fact, values of BAL leucocytes in BCD+PG06 and BCD+PG534 were not statistically different from those in the BCD group. In order to confirm these results, we further evaluate lung phagocytes populations by flow cytometry. As shown in Fig 2D, the number of CD11c^+^F4/80^+^MHCII^+^ macrophages was significantly higher in BCD+PG05-treated mice than in the other experimental groups. The numbers of lung CD45^+^Gr1^+^ and CD45^+^Gr1^high^ neutrophils was higher in BCD+PG05 than in BCD and BCD+PG534 groups (Fig 2E). Interestingly, lung CD45^+^Gr1^+^ and CD45^+^Gr1^low^ neutrophils in the BCD+PG06 group were no statistically different from BCD+PG05.

**Fig 3:**
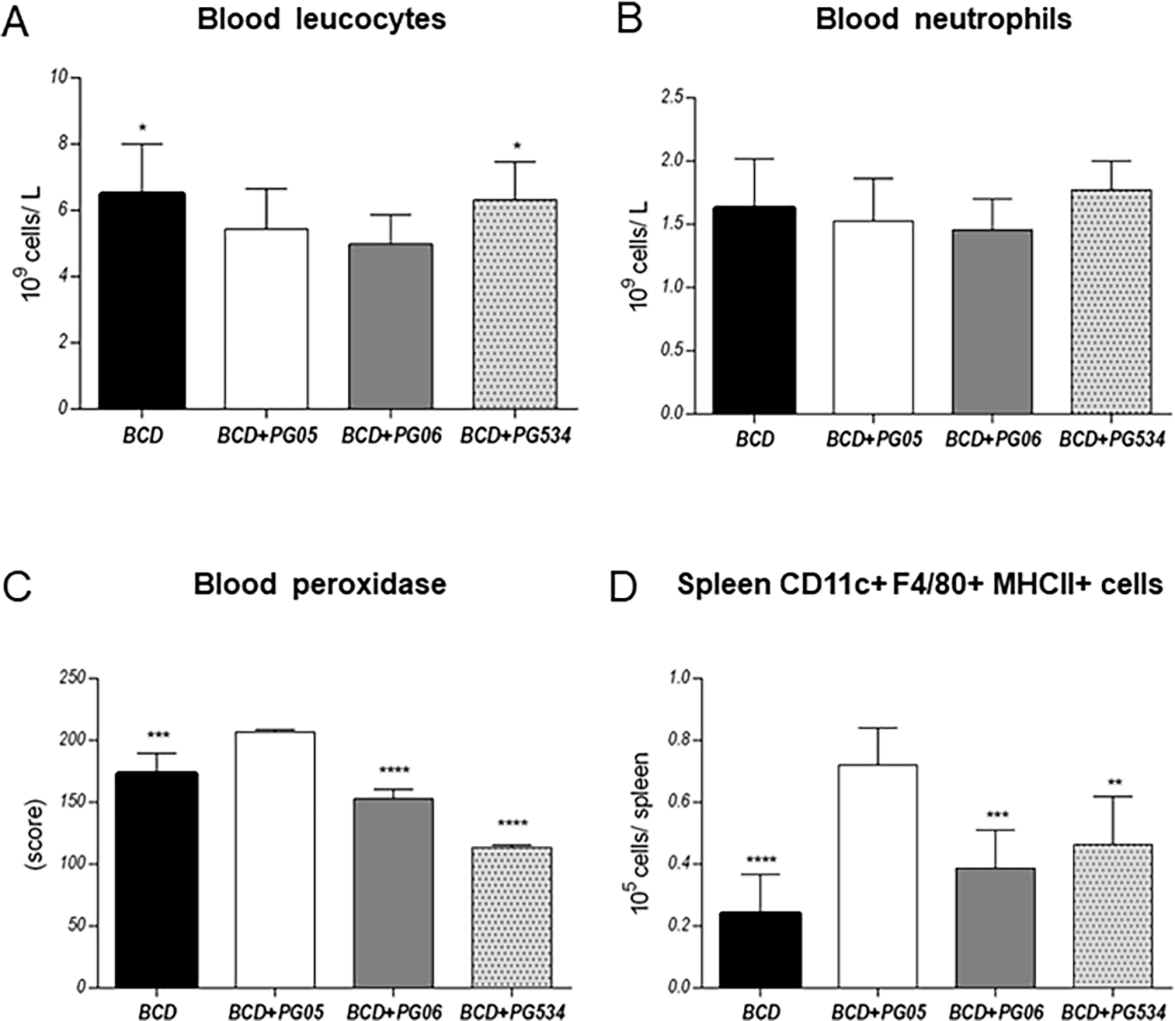
Effect of lactobacilli peptidoglycans on the systemic innate immune response to *Streptococcus pneumoniae* infection in malnourished mice. Immunocompromised-malnourished mice were replete for 7 days with a balanced conventional diet (BCD) or BCD supplemented with peptidoglycans from *Lactobacillus rhamnosus* CRL1505 (BCD+PG05), *L*. *plantarum* CRL1506 (BCD+PG06) or *L*. *rhamnosus* CRL534 (BCD+PG534), and then challenged with *S*. *pneumoniae*. The systemic innate immune response was studied two days after the pneumococcal challenge. (A) Number of blood leukocytes. (B) Number of blood neutrophils. (C) Score of blood peroxidase. (D) Number of spleen macrophages (CD11c^+^F480^+^MHCII^+^). Results represent data from three independent experiments. Results are expressed as mean ± SD. Asterisks represent statistical differences compared with BCD+PG05 group. * p < 0.05, ** p < 0.005, ***/**** p ≤ 0.001.

When we evaluated blood leucocytes, we found that infection increased the numbers of blood leucocytes (Fig 3A) and neutrophils (Fig 3B) as well as blood peroxidase activity (Fig 3C) in all the experimental groups. The number of blood leucocytes in BCD and BCD+PG534 groups were higher than in BCD+PG05, while no differences were observed with BCD+PG06. Taking into consideration that activated neutrophils show a high peroxidase activity in their granules [23], we decided to study peroxidase positive cells at day two post-infection. While the numbers of neutrophils from the studied groups were not significantly different between them (Fig 3B), the peroxidase activity of those immune cells was significantly higher in BCD+PG05-treated mice when compared to the other experimental groups (Fig 3C).

Spleen macrophages contribute significantly to early protection against bacterial dissemination [24]. Then we also evaluated the numbers of macrophages in spleen that were defined as CD11c+F4/80+MHCII+ cells. Results showed that spleen macrophages were significantly higher in BCD+PG05-treated mice than in the other groups (Fig 3D).

**Fig 4:**
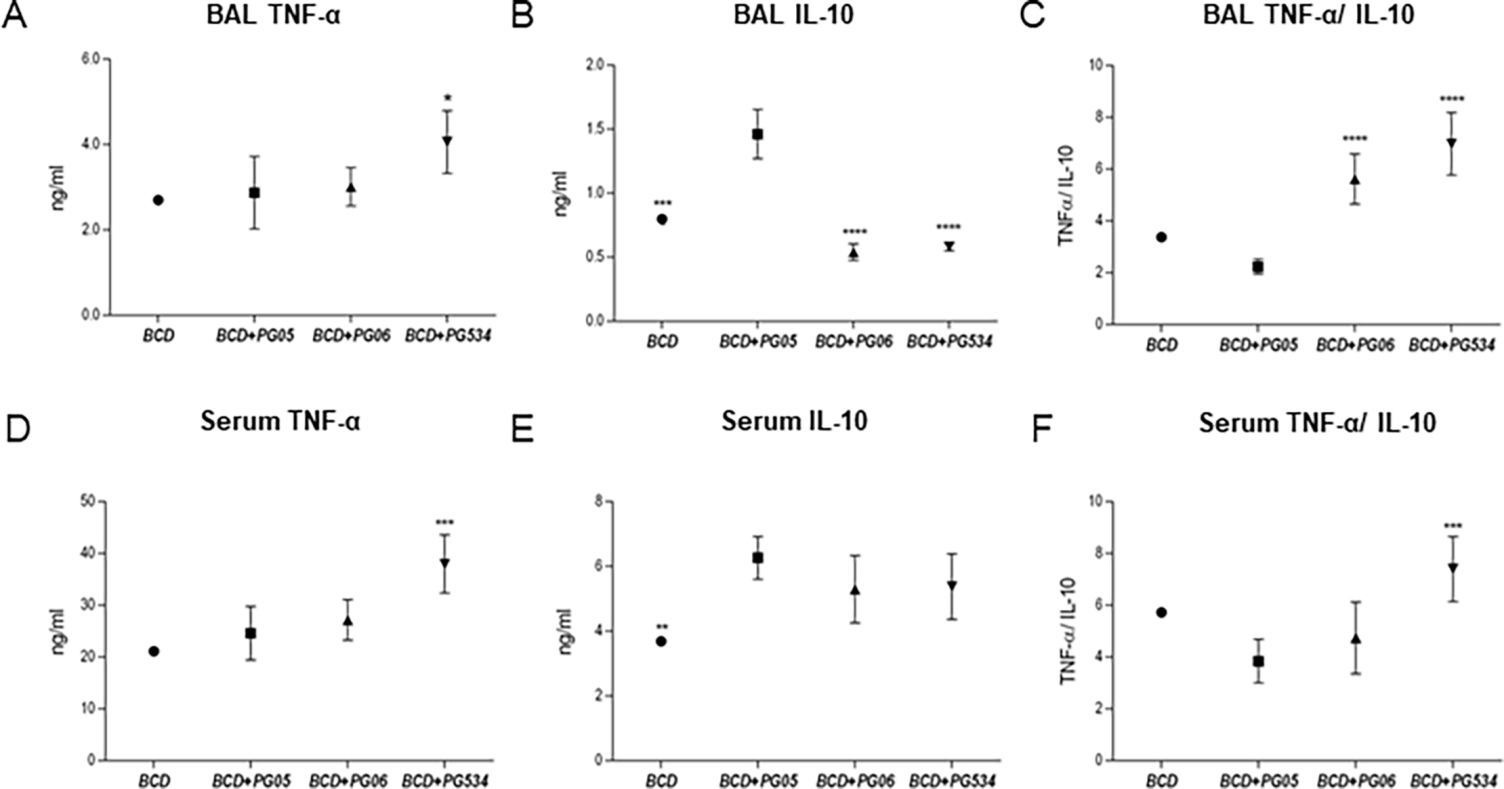
Effect of lactobacilli peptidoglycans on TNF-α and IL-10 production after *Streptococcus pneumoniae* infection in malnourished mice. Immunocompromised-malnourished mice were replete for 7 days with a balanced conventional diet (BCD) or BCD supplemented with peptidoglycans from *Lactobacillus rhamnosus* CRL1505 (BCD+PG05), *L*. *plantarum* CRL1506 (BCD+PG06) or *L*. *rhamnosus* CRL534 (BCD+PG534), and then challenged with *S*. *pneumoniae*. The levels of cytokines were studied two days after the pneumococcal challenge. (A) TNF-α, (B) IL-10, and (C) TNF-α/IL-10 ratio in broncho-alveolar lavages (BAL). (D) TNF-α, (E) IL-10, and (F) TNF-α/IL-10 ratio in serum. Results are expressed as mean ± SD. Asterisks represent statistical differences compared with BCD+PG05 group. * p < 0.05, ** p < 0.005, ***/**** p ≤ 0.001.

In addition, relevant cytokines involved in the innate immune response against *S*. *pneumoniae* were measured on BAL and blood (Fig 4). Levels of both TNF-α and IL-10 were increased after the challenge with *S*. *pneumoniae* in BAL and blood. BCD+PG05 group showed the highest values of BAL and blood IL-10 (Fig 4B and 4E) and the lowest TNF-α/IL-10 ratio (Fig 4C and 4F) when compared with the others experimental groups. Conversely, the BCD+PG534 group exhibited significant higher levels of BAL and blood TNF-α that the other groups (Fig 4A and 4D), while BCD+PG06 was not different from the BCD group.

### Effect of lactobacilli peptidoglycans on T cells immune response against pneumococcal infection in malnourished mice

**Fig 5:**
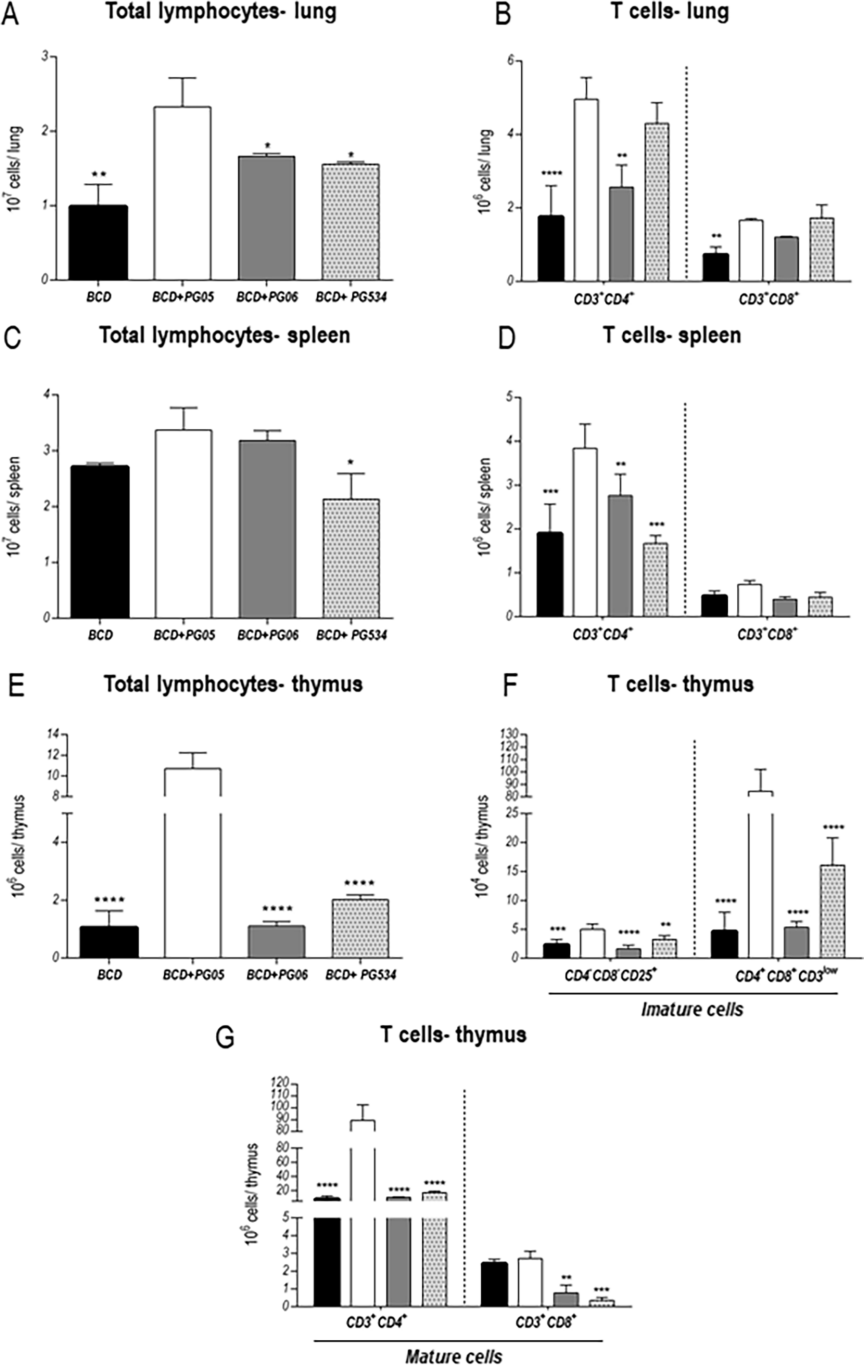
Effect of lactobacilli peptidoglycans on lung, spleen and thymus T cells numbers after *Streptococcus pneumoniae* infection in malnourished mice. Immunocompromised-malnourished mice were replete for 7 days with a balanced conventional diet (BCD) or BCD supplemented with peptidoglycans from *Lactobacillus rhamnosus* CRL1505 (BCD+PG05), *L*. *plantarum* CRL1506 (BCD+PG06) or *L*. *rhamnosus* CRL534 (BCD+PG534), and then challenged with *S*. *pneumoniae*. The numbers of T cells were studied ten days after the pneumococcal challenge. (A) Number of total lymphocytes in lung. (B) Number of CD3^+^CD4^+^ and CD3^+^CD8^+^ T cells in lung. (C) Number of total lymphocytes in spleen. (D) Number of CD3^+^CD4^+^ and CD3^+^CD8^+^ T cells in spleen. (E) Number of total lymphocytes in thymus. (F) CD4^-^CD8^-^CD25^+^ and CD4^+^CD8^+^CD3^low^ (immature cells). (G) CD3^+^CD4^+^ and CD3^+^CD8^+^ (mature cells). Results are expressed as mean ± SD. Asterisks represent statistical differences compared with BCD+PG05 group. * p < 0.05, **p < 0.005, ***/**** p ≤ 0.001.

Considering that the efficient clearance of *S*. *pneumoniae* depends on both cellular and humoral immunity, we evaluate the effect of peptidoglycans treatments on the number and function of effector cells and mediators of adaptive immune response against to this respiratory pathogen. Different populations of T cells were analyzed in lung, spleen, and thymus (Fig 5) at day 10 post-infection by flow cytometry. Lung lymphocytes were significantly higher in BCD+PG05-treated mice when compared with the other experimental groups (Fig 5A). In particular, increased numbers of lung CD3+CD4+ T cells were observed in BCD+PG05-treated animals when compared with BCD and BCD+PG06 groups. BCD control group showed lower values of CD3+CD8+ T cells compared with BCD+PG05 mice (Fig 5B). Worthy of note, although the total number of lung lymphocytes in BCD+PG534 group were different from BCD+PG05 mice, lung CD3+CD4+ and CD3+CD8+ T cells reached the values of the BCD+PG05 group. In addition, it was observed that spleen CD3+CD4+ T cells were significantly higher in BCD+PG05-treated mice when compared with the other experimental groups while no differences were found in spleen CD3+CD8+ T cells (Fig 5C and 5D).

When the total number of lymphocytes and the different subpopulation of T cells were evaluated in the thymus, we observed that BCD+PG05 mice had significantly higher numbers of total lymphocytes (Fig 5E) as well as double negative (DN) CD3^-^CD4^-^CD8^-^CD25^+^, double positive (DP) CD4^+^CD8^+^CD3^low^ (immature cells) (Fig 5F), and simple positive (SP) CD3^+^CD4^+^ T cells that the other experimental groups (Fig 5G). SP CD3^+^CD8^+^ T cells (Fig 5G) in BCD+PG05 were not different from BCD mice. However, levels of SP CD3^+^CD8^+^ T cells in the thymus of BCD+PG06 and BCD+PG534 were lower than the BCD+PG05 group (Fig 5G). In addition, it was observed that CD3^high^TCRαβ^+^ cells were higher in mice treated with BCD plus peptidoglycans when compared to those in the BCD group.

**Fig 6:**
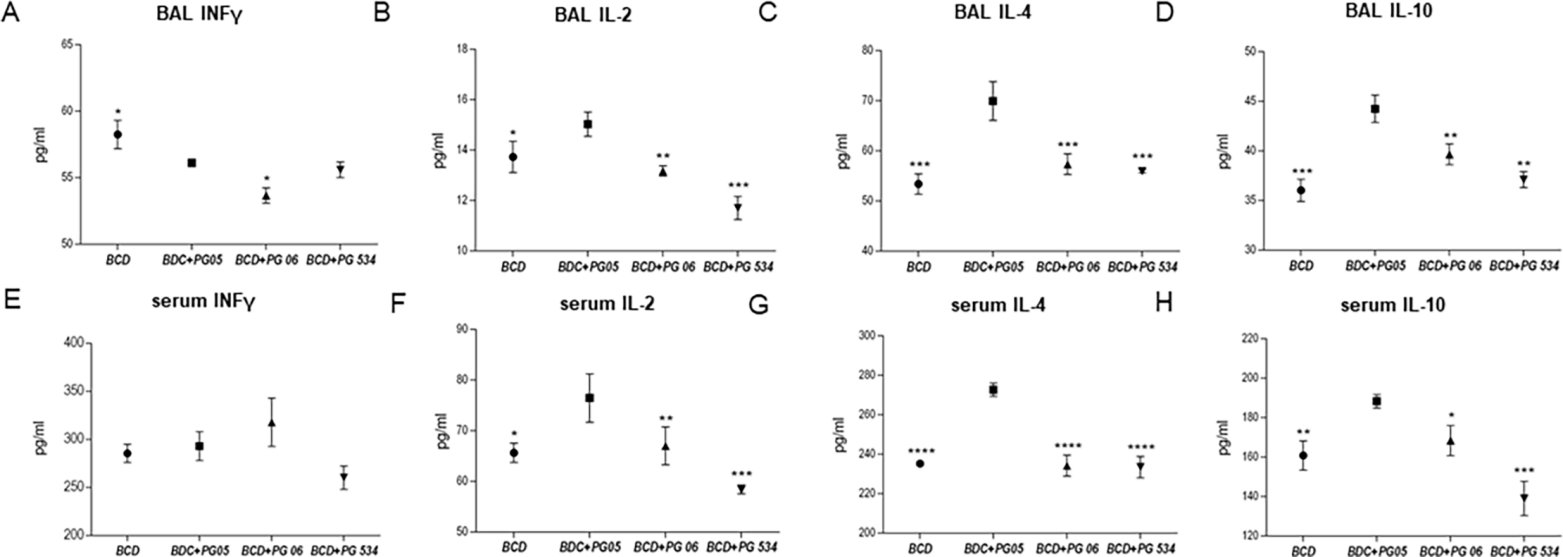
Effect of lactobacilli peptidoglycans on Th1 and Th2 cytokines production after *Streptococcus pneumoniae* infection in malnourished mice. Immunocompromised-malnourished mice were replete for 7 days with a balanced conventional diet (BCD) or BCD supplemented with peptidoglycans from *Lactobacillus rhamnosus* CRL1505 (BCD+PG05), *L*. *plantarum* CRL1506 (BCD+PG06) or *L*. *rhamnosus* CRL534 (BCD+PG534), and then challenged with *S*. *pneumoniae*. The levels of cytokines were studied ten days after the pneumococcal challenge. (A-D) Broncho-alveolar lavages (BAL), and (E-H) serum concentration of INF-γ, IL-2, IL-4, and IL-10. Results represent data from three independent experiments. Results are expressed as mean ± SD. Asterisks represent statistical differences compared with BCD+PG05 group. * p < 0.05, ** p < 0.005, ***/**** p ≤ 0.001.

We also evaluated Th1 and Th2 cytokines profiles in respiratory and systemic compartments at day 10 after challenge with *S*. *pneumoniae*. Levels of BAL and serum IFN-γ, IL-2, IL-4 and IL-10 increased significantly in all the experimental groups after challenge. However, BAL IFN-γ production was lower in groups treated with BCD plus peptidoglycans (Fig 6A), while serum IFN-γ did not show statistical differences between groups (Fig 6B). Levels of BAL and serum IL-2 were significantly higher in BCD+PG05-treated mice when compared with the other experimental groups (Fig 6B and 6F). In addition, repletion of malnourished mice with BCD+PG05 significantly improved the production of BAL and serum IL-4 and IL-10, which showed higher levels than those of the BCD control group (Fig 6C, 6D, 6G and 6H). Levels of these two cytokines in BCD+PG06-treated mice were not different from the BCD group, while serum IL-10 in BCD+PG534 mice was significantly lower when compared to the other experimental groups (Fig 6D and 6H).

### Effect of lactobacilli peptidoglycans on humoral immune response against pneumococcal infection in malnourished mice

**Fig 7:**
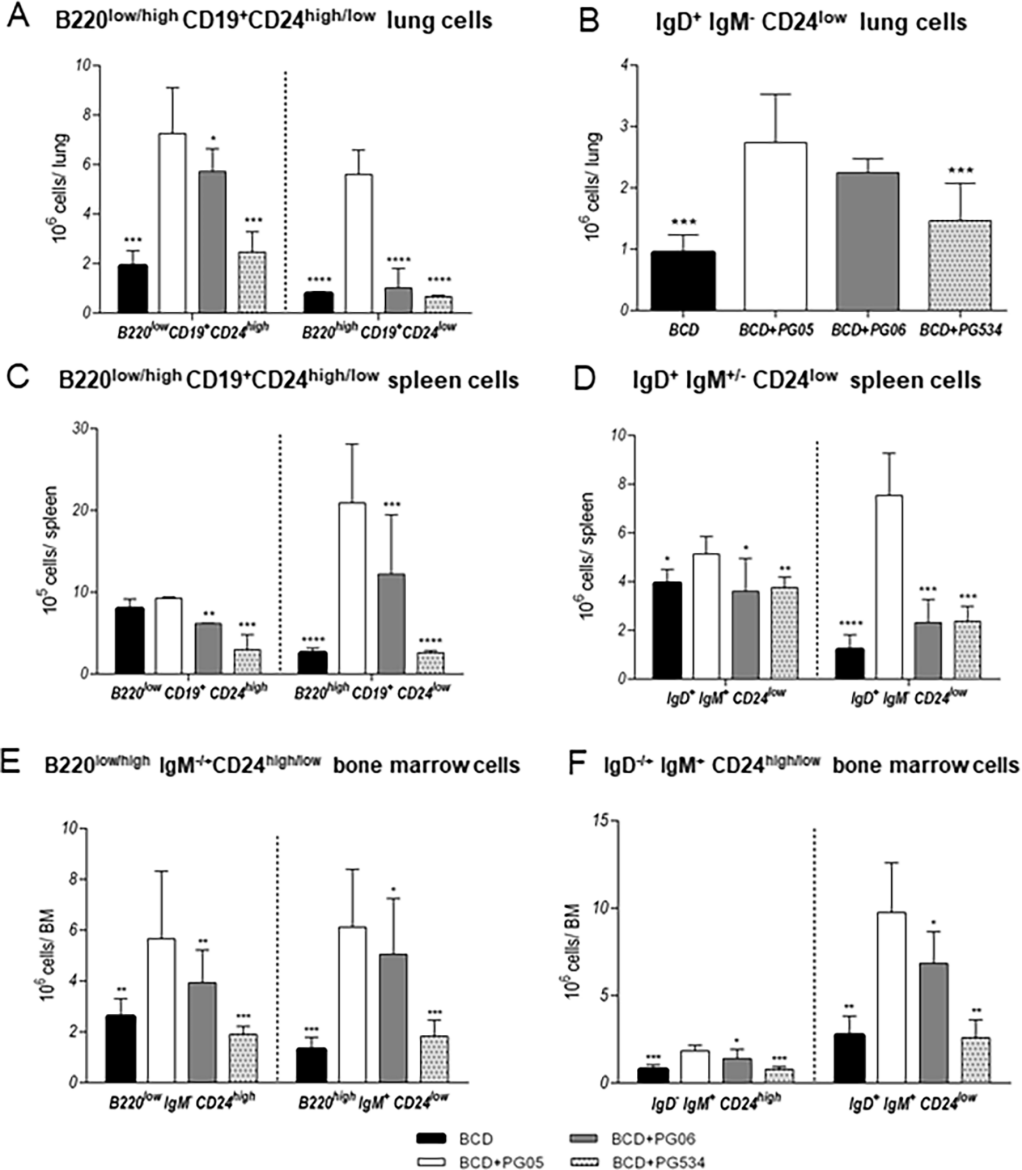
Effect of lactobacilli peptidoglycans on lung, spleen and bone marrow (BM) B cells numbers after *Streptococcus pneumoniae* infection in malnourished mice. Immunocompromised-malnourished mice were replete for 7 days with a balanced conventional diet (BCD) or BCD supplemented with peptidoglycans from *Lactobacillus rhamnosus* CRL1505 (BCD+PG05), *L*. *plantarum* CRL1506 (BCD+PG06) or *L*. *rhamnosus* CRL534 (BCD+PG534), and then challenged with *S*. *pneumoniae*. The numbers of B cells were studied fifteen days after the pneumococcal challenge. Different lung B cells subpopulations: (A) B220^low^CD19^+^CD24^high^ (immature cells) and B220^high^CD19^+^CD24^low^ (mature cells) (B) IgD^+^IgM^-^CD24^low^ (mature cells) Different spleen B cells subpopulations: (C) B220^low^CD19^+^CD24^high^ (immature cells) and B220^high^CD19^+^CD24^low^ (mature cells). (D) IgD^+^IgM^+^CD24^low^ (immature cells) and IgD^+^IgM^-^CD24^low^ (mature cells). Different B cells subpopulations in BM: (E) B220^low^IgM^-^CD24^high^ pre-pro B cells and B220^high^IgM^+^CD24^low^ mature B cells. (F) IgD^-^IgM^+^CD24^high^ immature B cells and IgD^+^IgM^+^CD24^low^ mature B cells. Results represent data from three independent experiments. Results are expressed as mean ± SD. Asterisks represent statistical differences compared with BCD+PG05 group. * p < 0.05, ** p < 0.005, ***/**** p ≤ 0.001.

We studied the humoral immune response to pneumococcal infection by evaluating B cells in different organs and the levels and activity of specific antibodies. The analysis of B cells on distinct maturation states was performed on day 15 post-infection (Fig 7). We observed that BCD+PG05 mice showed higher values of lung total lymphocytes compared with the other groups (Fig 5A). In addition, BCD+PG05 group showed significantly increased numbers of B220^low^CD19^+^CD24^high^ immature, B220^high^CD19^+^CD24^low^ (Fig 7A), and IgD^+^IgM^-^CD24^low^ mature B cells (Fig 7B) in lungs when compared to the other experimental groups (Fig 9A). B220^high^CD19^+^CD24^low^ and IgD^+^IgM^+/-^CD24^low^ mature B cells in spleen were also higher in BCD+PG05-treated mice than in the other groups (Fig 7C and 7D).

In addition, we evaluated B cells populations in bone marrow. Bone marrow total lymphocytes in BCD+PG05 mice were higher than the other experimental groups. In addition, BCD+PG05 group exhibited an increase of B220^low^IgM^-^CD24^high^ pro-pre (Fig 7E), and IgD^-^IgM^+^CD24^high^ immature (Fig F) B cells when these cells were compared with the BCD, BCD+PG06 and BCD+PG534 groups. B220^high^IgM^+^CD24^low^ and IgD^+^IgM^+^CD24^low^ mature B cells in BCD+PG05-treated mice were also significantly higher than those observed in the other groups (Fig 7E and 7F).

**Fig 8:**
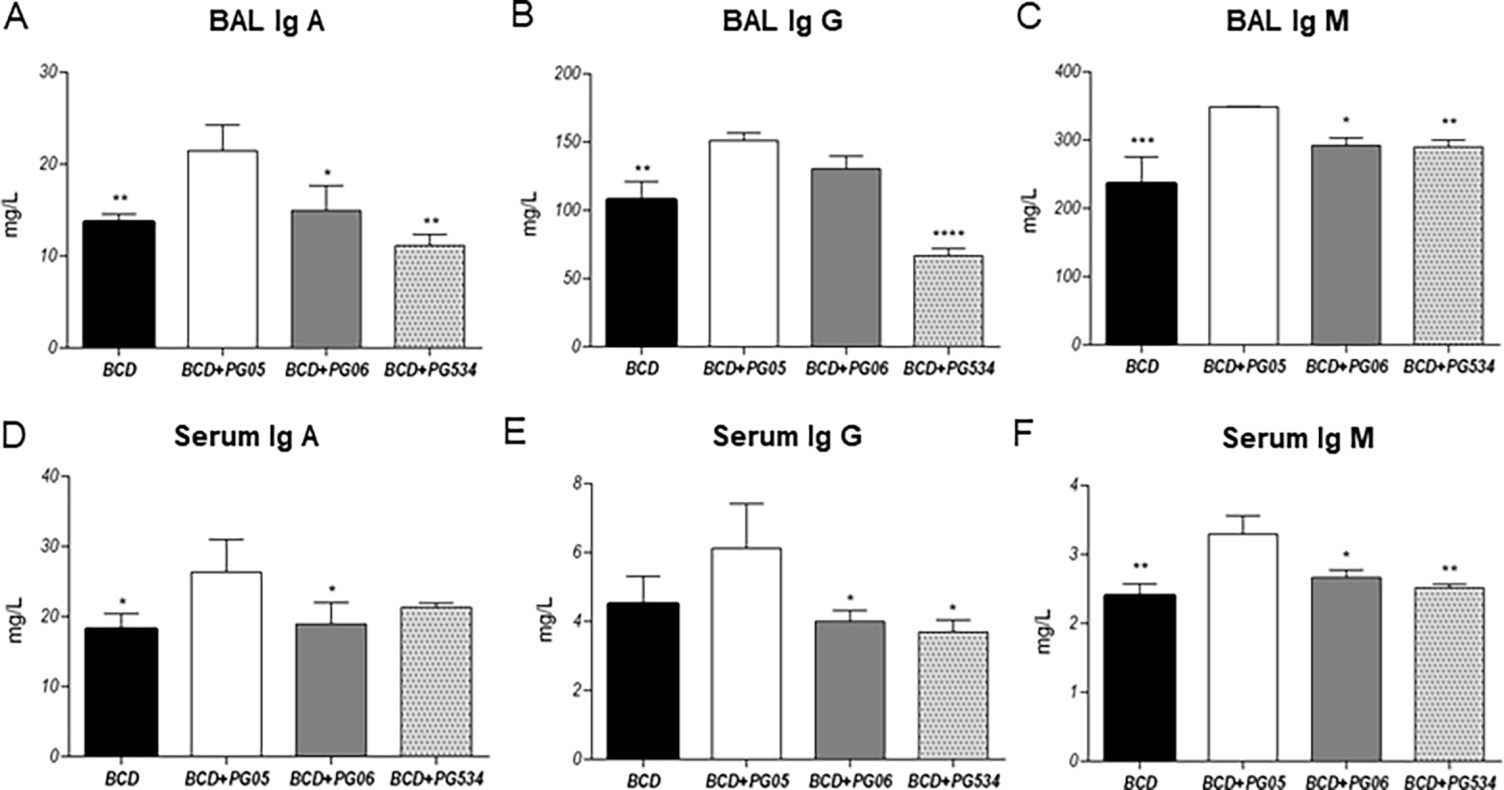
Effect of lactobacilli peptidoglycans on anti-pneumococcal antibodies levels after *Streptococcus pneumoniae* infection in malnourished mice. Immunocompromised-malnourished mice were replete for 7 days with a balanced conventional diet (BCD) or BCD supplemented with peptidoglycans from *Lactobacillus rhamnosus* CRL1505 (BCD+PG05), *L*. *plantarum* CRL1506 (BCD+PG06) or *L*. *rhamnosus* CRL534 (BCD+PG534), and then challenged with *S*. *pneumoniae*. The levels of antibodies were studied fifteen days after the pneumococcal challenge. (A) Levels of anti-peumococcal IgA, (B) IgG and (C) IgM in broncho-alveolar lavages. (D) Levels of anti-peumococcal IgA, (E) IgG and (F) IgM in serum. Results represent data from three independent experiments. Results are expressed as mean ± SD. Asterisks represent statistical differences compared with BCD+PG05 group. * p < 0.05, **p < 0.005, ***/**** p ≤ 0.001.

**Fig 9:**
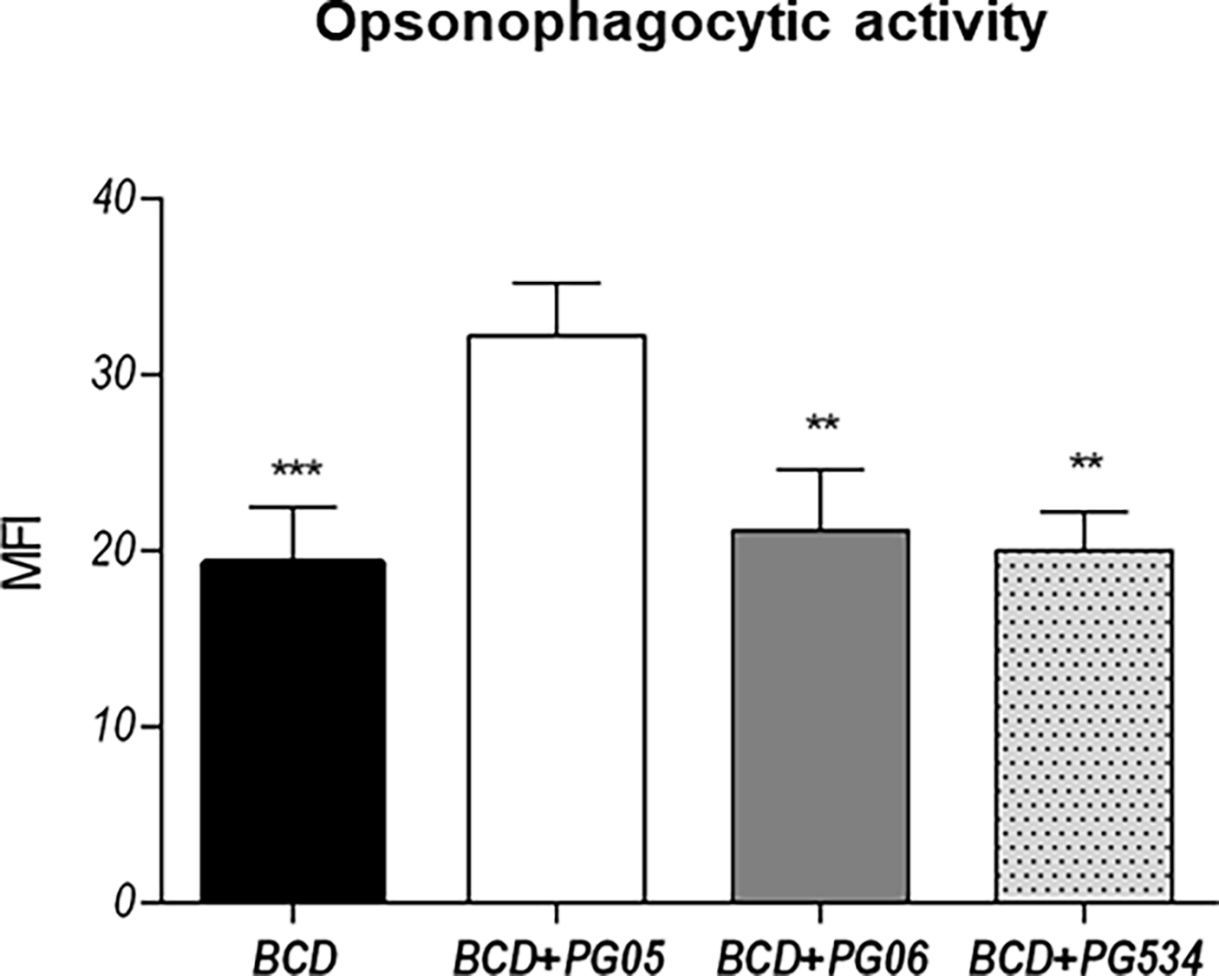
Effect of lactobacilli peptidoglycans on the opsonophagocytic activity of anti-pneumococcal antibodies after *Streptococcus pneumoniae* infection in malnourished mice. Immunocompromised-malnourished mice were replete for 7 days with a balanced conventional diet (BCD) or BCD supplemented with peptidoglycans from *Lactobacillus rhamnosus* CRL1505 (BCD+PG05), *L*. *plantarum* CRL1506 (BCD+PG06) or *L*. *rhamnosus* CRL534 (BCD+PG534), and then challenged with *S*. *pneumoniae*. The opsonophagocytic activity of antibodies were studied fifteen days after the pneumococcal challenge. Results represent data from three independent experiments. Results are expressed as mean ± SD. Asterisks represent statistical differences compared with BCD+PG05 group. * p < 0.05, ** p < 0.005, *** p ≤ 0.001.

Finally, we determined the levels of anti-pneumococcal antibodies in serum and BAL (Fig 8). Concentrations of specific IgA, IgG, and IgM in both serum and BAL were significantly higher in mice that received PG05 when compared to the BCD group. BAL and serum IgA and IgM antibodies in BCD+PG06-treated mice were not different from the BCD group, while BAL IgG levels were similar to those observed in the BCD+PG05 group (Fig 8). In addition, all the studied antibodies in the BCD+PG534 group were similar to BCD mice (Fig 8). We also evaluated the opsonophagocytic activity of serum anti-pneumococcal antibodies of each group of mice, and found that the capacity of antibodies from BCD+PG05 mice to increase the phagocytosis of heat-killed pneumococci was significantly higher when compared to the other experimental groups (Fig 9).

## Discussion

In recent years, there have been substantial advances in the knowledge of the molecular crosstalk between beneficial microbes and the mucosal immune system through the identification of bacterial molecules and hosts receptors involved in immunomodulation. In this regard, *L*. *rhamnosus* CRL1505 is an immunobiotic strain that is used as a probiotic specially directed to children [25], and has been widely characterized by our laboratory [10–13,15,22,26]. Our studies have demonstrated that the supplementation of BCD with nasally administered viable CRL1505 strain, during the recovery of malnourished mice, allows an improvement of the respiratory immune response and emergency granulopoiesis [10–12].

Of note, the use of molecules from beneficial microbes could be an interesting alternative to stimulate immunity in immunocompromised hosts, in which viable microorganisms could involve a health risk [27]. In this regard, we have identified that the peptidoglycan is an important bacterial component for the immunomodulatory activities of *L*. *rhamnosus* CRL1505 and we have focused our attention on the immunomodulatory effect of this peptidoglycan in immunocompromised hosts. Of interest, we have shown that PG05 was as effective as the viable bacteria for improving systemic and respiratory innate immune responses during recovery of immunocompromised-malnourished mice [15]. Here, we have further advanced in the study of PG05 by evaluating whether its immunoregulatory activity is a strain specific property. We performed comparative studies to evaluate the effect of peptidoglycans from different lactobacilli on the innate and adaptive immune responses to pneumococcal infection in malnourished mice. We demonstrated that PG05 has unique immunomodulatory capacities that cannot be extrapolated to peptidoglycans from other lactobacilli of the same species or lactobacilli with immunomodulatory properties.

We clearly demonstrated that PG05, nasally administered during the repletion treatment of malnourished mice, was the only peptidoglycan able to increase the resistance against *S*. *pneumoniae* infection, which was demonstrated by the lower lung pathogen counts accompanied by reduced lung damage. These results are in line with previous work [15]. Nevertheless, this effect was not observed in the animals that received PGs extracted from *L*. *rhamnosus* CRL534, a non-probiotic strain [28], or *L*. *plantarum* CRL1506, whose nasal administration modulates the antiviral immune response in the lung [29]. To explain the differences in the immunomodulatory activities of the studied PGs one important point should be considered. It is a well-established fact that the immunomodulatory properties of probiotic bacteria are strain specific. In this sense, several examples can be found in scientific published works regarding this statement for immunobiotic lactobacilli [30,31]. However, the PG may be one of the different bacterial molecules associated to their beneficial effects. Thus, our studies were conducted to elucidate the immunological mechanisms that allow us to understand the different effects of PGs under study.

It is known that during lung infections such as those produced by *S*. *pneumoniae*, phagocytic cells are key in host defense [32,33]. When the bacterial inoculum increases above the level at which alveolar macrophages can contain bacteria without recruitment of further phagocytes, cross-talk between macrophages, epithelial cells and T cells results in the release of chemokines and recruitment of further leukocytes, particularly polymorphonuclear cells. These cells become critical effectors of bacterial clearance [34]. For this reason, the low number of lung phagocytic cells in malnourished mice could be related to impaired host defenses, which would explain their difficulties to eradicate the pathogens from the lungs as we showed previously [10,11]. Our previous studies [15] and the present results showed that PG05 is able to beneficially modulate the balance between TNF-α and IL-10 during innate response, allowing an improved clearance of *S*. *pneumoniae* and reducing the inflammatory lung tissue damage at the same time. We found that the TNF-α/IL-10 ratio of BCD+PG06 and BCD+PG534 mice was higher than BCD+PG05 mice, because PG06 does not induce IL-10 production and PG534 induce higher levels of TNF-α compared with BCD+PG05. These findings had a good correlation with lung histopathological studies that revealed significantly higher infiltration of inflammatory cells and tissue damage in the lungs of these groups when compared to the BCD+PG05 and BCD groups. We speculate that other cytokines and chemokines of innate response, as IL-1β, KC, MCP-1 [35–37], produced by lung epithelial cell or alveolar macrophages would be involved. Herrera et al., [11] demonstrated that the CRL1505 strain was able to modulate the CXCL12/CXCR4 axis. Even more, this cytokine balance induced by PG05 is also responsible for the increase of lung phagocytic cell number, but also the blood peroxidase activity and the spleen macrophages number.

Pneumococcal exposure can lead to the generation of both T cell and B cell immune responses to polysaccharide and protein antigens [38,39]. In this work, we evaluate the effect of PGs treatments on the number and function of effector cells and mediators of adaptive immune response against to this respiratory pathogen. Malnutrition severely affects T population in the bone marrow, thymus, spleen and lung [12,22,40]. The recovery of T cells is important for the protection of the host through the production of divers cytokines that control and coordinate several immune effector mechanisms and their capacity to influencing antibody production by B cells. In this way, the nasal administration of the CRL1505 strain reduces quantitative and qualitative alterations of CD4 T cells in recovering malnourished mice [10]. In the current study, BCD+PG05 mice showed a higher number of double negative CD25+ and double positive T cells after infection than those of others groups, indicating an active thymopoiesis [41]. These findings are probably related to the increase of CD4 simple positive cells, observed only in this group. In addition, to promoting the development of CD4 T cells in the thymus after the pneumococcal challenge, the PG05-treatment managed to increase the spleen CD3+CD4+ population, and maintain the values of lung CD3+CD4+cells higher than the other groups. This effect of PG05 agree with the effects induced by viable *L*. *rhamnosus* CRL1505 and could be key to the protection against *S*. *pneumoniae* infection [12,42].

Additionally, only the PG05 treatment was able to modulate cytokine production in response to respiratory *S*. *pneumoniae* challenge. It is known that under inflammatory conditions, cytokines in the airways environment change dramatically. When a Th2 response is needed, there is a production of IL-4, IL-5, and IL-6, which contributes to stimulate B cells to proliferate and mature into antibody producing cells [43]. Here we found a significant up-regulation in BAL and serum Th2-cytokine IL-4 in malnourished mice fed with BCD+PG05 after pneumococcal infection, unlike PG06 or PG534 treatments. This is consistent with the enhancement of BAL and serum IgA, IgG and IgM anti-pneumococcal production observed in this group and, is in line with previous reports [10,44]. On the other hand, PG05 treatment induced the greatest increase of IL-10 in BAL after pneumococcal infection, which could be associated to the reduction of tissue damage and recruitment of neutrophils to the airways observed in this group [45]. Even more, only PG05 induced production of IL-2, which participates in CD4+ T-cell response against pneumococcal antigens [46].

Regarding the impairment of the humoral immune response in malnourished hosts mentioned above, we demonstrated that protein malnutrition remarkably reduces BAL and serum anti-pneumococcal antibodies [8,10]. Moreover, the opsonophagocytic activity of IgG antibodies was significantly reduced in malnourished mice [10]. These findings are associated with the impairment of B cell population in bone marrow without affecting their capacity to produce antibodies [10,22]. In the present work, we demonstrated that malnourished mice treated with PG05 were able to mount a normal immune response against the respiratory infection. In fact, the increase of bone marrow and lung immature and mature B cells, and splenic mature B lymphocytes were found in the BCD+PG05 group. These results are associated with the highest local and systemic anti-pneumococcal antibody production induced with BDC+PG05 group. Moreover, the PG extracted from probiotic CRL1506 strain induced an increase of lung mature B cells, which is related with the levels increased of IgG in BAL. Our results also showed that BCD+PG534 treatment had no beneficial effect on number or activity of B cells.

In this point, it is necessary to emphasize that PG06 would not be involved in the immunomodulatory properties of viable *L*. *plantarum* CRL1506. This finding is in line with our previous observations. As it was mentioned above, nasally administered viable CRL1506 and CRL1505 strains improve protection against RSV infection. However, when lactobacilli were heat-treated, only *L*. *rhamnosus* CRL1505 retained its capacity to improve respiratory antiviral immunity [29]. This observation indicates that viability is a necessary condition for the beneficial effects of *L*. *plantarum* CRL1506 that cannot be obtained with non-viable lactobacilli or its peptidoglycan. Another key difference in immunomodulatory ability of *L*. *plantarum* CRL1506 and *L*. *rhamnosus* CRL1505, is the capacity of the CRL1505 strain, when orally administered, to improve immunity in distal mucosal sites such as the respiratory tract, an effect that is not achieved by the CRL1506 strain [26,47]. Some research works demonstrated that the gut microbiome provide signals to sustain immune defense mechanisms in the respiratory tract allowing efficient effector responses upon challenge by pathogens [48,49]. It has been proposed as a mechanism for distal modulation that respiratory and peripheral immune cells are directly exposed to bacterial products released in the gut. There is evidence that bacterial molecules from gut microorganisms such as peptidoglycan can be absorbed and circulate throughout the host allowing the modulation of distal immune cells [49]. In this regard, Ichinohe et al. [48] showed that bacterial products from gut commensals stimulate immune cells systemically and that factors released by those cells improve the immune response to influenza virus infection. Therefore, it is likely to speculate that when *L*. *plantarum* CRL1506 and *L*. *rhamnosus* CRL1505 are orally administered, the adverse conditions of the gastrointestinal tract induce the release of bacterial molecules including peptidoglycan that can be absorbed, circulate, and influence in distal immune cells. However, because PG05 preserves the immunomodulatory properties of viable bacteria while PG06 does not, only *L*. *rhamnosus* CRL1505 influence in distal mucosal sites. This hypothesis opens an interesting topic for future investigations.

### Conclusions

In the present study, we demonstrated that dietary recovery of malnourished mice with nasal administration of peptidoglycan from *L*. *rhamnosus* CRL1505 improves not only the innate immune response but the respiratory and systemic adaptive immune response as well. Then, we demonstrated for the first time that peptidoglycan from *L*. *rhamnosus* CRL1505 preserves the complete immunomodulatory properties of viable bacteria. In addition, by performing comparative studies with the peptidoglycan from *L*. *plantarum* CRL1506, an immunomodulatory bacterium, we consider that peptidoglycan from *L*. *rhamnosus* CRL1505 has unique immunomodulatory properties that could not be extended to peptidoglycans from other immunobiotic strains. The knowledge of the molecular characteristics of PG05 is indispensable to understand immunomodulatory abilities of *L*. *rhamnosus* CRL1505.
